# Identification of prognostic biomarkers for nasopharyngeal carcinoma through microRNA‑mRNA network analysis combined with screening of immune‑related genes

**DOI:** 10.1371/journal.pone.0361607

**Published:** 2026-10-29

**Authors:** Lihe He, Xuan Huang, Ping Huang

**Affiliations:** 1 Department of Oncology, Liuzhou Hospital of Traditional Chinese Medicine, Liuzhou, China; 2 Department of Gastroenterology, Liuzhou Hospital of Traditional Chinese Medicine, Liuzhou, China; Guangdong Medical University, CHINA

## Abstract

**Background:**

Nasopharyngeal carcinoma (NPC) is a common head and neck cancer, and radiotherapy is its main treatment. Recurrence and metastasis are the main causes of treatment failure. Although immunotherapy can prolong patient survival, the molecular mechanisms underlying NPC development and progression remain unclear. MicroRNAs (miRNAs) and their target genes are abnormally expressed in many tumors and are closely involved in tumor development. Therefore, this study aimed to identify key miRNAs and immune-related target genes in NPC, construct miRNA–mRNA regulatory networks, and screen for and validate core immune genes to identify prognostic molecular biomarkers for NPC.

**Methods:**

We downloaded NPC-related miRNA (GSE32960) and mRNA (GSE12452, GSE13597, GSE64634) microarray datasets from the Gene Expression Omnibus (GEO) and identified differentially expressed miRNAs (DEMs) and mRNAs (DEGs) using R. Target genes of DEMs were predicted with TargetScan, miRDB, and miRWalk, and then intersected with DEGs to identify core genes. A miRNA–mRNA regulatory network was constructed in Cytoscape. The core genes were further overlapped with immune-related gene sets, followed by functional enrichment analysis. A prognostic risk model was established using LASSO–Cox regression, and immune cell infiltration was evaluated with CIBERSORT. Model validation, immunohistochemical analysis, and drug–gene interaction analysis were also performed. In vitro, RT–qPCR was conducted in C666-1 and NP69 cell lines to verify the expression of key genes.

**Results:**

We identified 213 potential target genes of key miRNAs. Three miRNAs (hsa-miR-30e-5p, hsa-miR-30a-5p, and hsa-miR-20a-5p) were associated with NPC prognosis, and 17 core immune-related genes were enriched in cytokine-mediated signaling, chemokine response, and JAK-STAT pathways. Five genes (CXCL11, SOCS1, ROBO1, LGR5, and RORB) showed promising diagnostic value for NPC; their expression was positively correlated (P < 0.05) with CD8 ⁺ T-cell, macrophage, and neutrophil infiltration. Drug-gene analysis predicted that 5-fluorouracil targeted LGR5 and aldesleukin targeted SOCS1. RT-qPCR demonstrated that SOCS1 was upregulated approximately 3.7-fold in NPC cells (P < 0.01).

**Conclusion:**

This study identified potential novel key genes in nasopharyngeal carcinoma, providing new therapeutic targets for the treatment of this disease.

## 1. Background

Nasopharyngeal carcinoma (NPC) is among the most common malignant tumors of the head and neck. It arises from the mucosal epithelium of the nasopharynx, typically originating in the pharyngeal recess (fossa of Rosenmüller). NPC exhibits a distinct geographical distribution and is particularly prevalent in Asia, especially in southern China [1,2]. Radiotherapy is a primary treatment modality for NPC [2],however, despite significant advances in radiation techniques, the 5-year survival rate for patients with early-to late-stage disease treated with radiotherapy alone remains merely30%–50% [3], with recurrence and metastasis remaining the leading causes of treatment failure and poor prognosis. In recent years, the introduction of immunotherapy has extended patient survival modestly; nevertheless, a subset of patients still develop resistance to radiotherapy or chemotherapy, ultimately leading to disease progression or even death. Currently, durable and effective therapeutic strategies for advanced NPC are lacking. The malignant phenotype of tumors is often driven by underlying molecular abnormalities; the initiation, progression, and distant metastasis of NPC are closely associated with genetic alterations and dysregulation of relevant signaling pathways. Therefore, molecular and genetic aberrations serve not only as diagnostic and prognostic markers for NPC but also as promising targets for biological therapy.

MicroRNAs (miRNAs) are small endogenous non-coding RNAs, typically 22–25 nucleotides in length [4]. They regulate gene expression post-transcriptionally by directly binding to target mRNAs. Aberrant miRNA expression can drive carcinogenesis, and miRNAs can function as either tumor suppressors or oncogenes; their dysregulation is associated with disease prognosis and clinical manifestations [5–7]. Numerous studies have demonstrated that various miRNAs are aberrantly expressed in nasopharyngeal carcinoma (NPC) and modulate multiple signaling pathways, suggesting they play a critical role in the initiation and progression of this malignancy [8–10]. Aberrantly expressed miRNAs are closely associated with clinical stage, pathological differentiation, sensitivity to radiotherapy and chemotherapy, and long-term prognosis in NPC patients, highlighting their considerable potential as diagnostic biomarkers, prognostic indicators, and therapeutic targets [5]. However, the mechanisms by which miRNAs and their target genes drive NPC progression and poor patient prognosis remain poorly understood.

In recent years, immunotherapy—exemplified by programmed death receptor-1 (PD-1)/programmed death ligand-1 (PD-L1) immune checkpoint inhibitors—has demonstrated significant clinical efficacy in the treatment of recurrent/metastatic nasopharyngeal carcinoma (NPC). However, a substantial proportion of patients fail to respond to this treatment or develop resistance, underscoring the need for more precise immune-related biomarkers to identify those likely to benefit. Immune-related genes (IRGs) are extensively involved in multiple biological processes, including antigen presentation, T-cell activation, cytokine production, immune checkpoint regulation, and immune cell recruitment; their expression levels can be directly or indirectly regulated by miRNAs. For example, miR-155 targets PD-L1 expression to modulate immune suppression [11,12], while miR-34a influences regulatory T cell (Treg) recruitment by modulating the chemokine CCL22 [13]. Therefore, identifying key immune-related genes regulated by miRNAs through miRNA-mRNA network analysis holds promise for elucidating the upstream molecular mechanisms underlying immune evasion and prognostic differences in NPC.

In recent years, only a limited number of studies have focused on constructing competitive endogenous RNA (ceRNA) networks in nasopharyngeal carcinoma (NPC) and performing prognostic analyses. The majority of existing studies, however, have focused on the long non-coding RNA (lncRNA)–miRNA–mRNA tripartite network, whereas the development of prognostic models based on the miRNA–mRNA binary regulatory network remains relatively underexplored. Crucially, although NPC prognostic models incorporating immune-related genes have been reported, these models typically rely exclusively on mRNA expression data and neglect the post-transcriptional regulation of immune-related genes by upstream miRNAs. As a result, elucidating the underlying drivers of aberrant immune-related gene expression remains challenging, and miRNAs—molecules with potential therapeutic value—are excluded from the prognostic assessment system.

Therefore, this study characterized differentially expressed miRNAs and mRNAs in nasopharyngeal carcinoma (NPC) and conducted functional enrichment analysis to explore the potential biological regulatory mechanisms of candidate genes. We applied LASSO regression to construct a risk prediction model for NPC and assessed the diagnostic value and prognostic performance of key immune-related genes in a validation cohort. Furthermore, we analyzed the immune infiltration landscape of the NPC microenvironment to elucidate the intrinsic relationship between core immune-related genes and the tumor immune microenvironment (TIME). This study aims to investigate the potential roles of miRNA–mRNA regulatory networks in the molecular dysregulation of NPC, identify novel immune-related genes, and provide a theoretical basis for prognosis assessment and immunotherapy in NPC patients.

## 2. Materials and methods

### 2.1 Data sources

We downloaded NPC-related datasets GSE32960, GSE12452, GSE13597, and GSE64634 from the Gene Expression Omnibus (GEO) database (https://www.ncbi.nlm.nih.gov/gds). GSE32960 contains miRNA expression data, whereas GSE12452, GSE13597, and GSE64634 contain mRNA expression data. All datasets were acquired and processed using R software (version 4.3.1).

### 2.2 Data preprocessing and adjustment for batch effects

Given the heterogeneity resulting from different sequencing platforms and clinical samples, We merged and performed batch correction on the three mRNA microarray datasets using the limma package [14] in R, and employed the “sva” package [15] to remove batch effects. We then screened the datasets by: (1) excluding samples without survival time or survival status, (2) converting probes to gene symbols,(3) excluding probes that matched multiple genes,(4) calculating the average expression value for genes with multiple probes, (5) retaining only human samples for miRNA data.

### 2.3 miRNA target gene prediction and miRNA-mRNA network analysis

In this study, we predicted the target genes of the differentially expressed miRNAs using the TargetScan, miRDB, and miRWalk databases. A cutoff value >80 was used for the prediction score to screen for high-confidence results and reduce the false-positive rate.. After integrating the prediction results from the three databases, we intersected these results with a set of differentially expressed genes to identify key candidate genes for NPC. We subsequently used Cytoscape v3.6.0 software [16] to visualize the regulatory relationships between miRNAs and their target genes and design the miRNA-target gene regulatory network.

### 2.4 Identification of immune-related genes

We retrieved 1793 immune-related genes(IRGs) from the ImmPort database [17] (https://www.immport.org/home). A Venn diagram was generated using the VennDiagram package in R to determine the overlap between candidate target genes for NPC and IRGs. These overlapping genes were subsequently designated as core IRGs.

### 2.5 Gene ontology (GO) and Kyoto encyclopedia of genes and genomics (KEGG) enrichment analysis

We utilized the ClusterProfiler package in R to perform GO and KEGG enrichment analyses on the core immune-related genes identified via screening [18–20], setting the false discovery rate (FDR) threshold at <0.05.

### 2.6 Screening of prognostic biomarkers and model development

Using gene expression profiles and clinical survival data from the Cancer Genome Atlas-head and neck squamous cell carcinoma (TCGA-HNSC) dataset, we employed least absolute shrinkage and selection operator (LASSO)–Cox regression analysis to identify prognostic genes and design a risk-scoring model. The optimal (λ) value was determined through 10-fold cross-validation, key prognostic genes were further identified, and risk scores for each sample were calculated based on the obtained regression coefficients. The median risk score was used as the cutoff value to divide the patient cohort into high- and low-risk groups. Subsequently, risk score distribution plots, survival status scatter plots, and gene expression heat maps were generated to visualize the characteristics of the model. Moreover, Kaplan–Meier survival curves were generated, and the log-rank test was used to assess the statistical significance of survival differences between groups. Time-dependent receiver operating characteristic (ROC) curves were designed to evaluate the model’s predictive performance for 1-, 3-, and 5-year overall survival (OS), with the area under the curve (AUC) with 95% confidence interval (CI) serving as the primary evaluation metric. The UALCAN online platform (University of Alabama at Birmingham cancer data analysis portal) was used to analyze gene expression levels in TCGA-HNSC cohort, and the Human Protein Atlas database was used to obtain immunohistochemical images of the key candidate genes (RORB, LGR5, ROBO1, SOCS1, and CXCL11) in normal nasopharyngeal and head and neck cancer tissues. Differences in protein expression levels and staining intensity between normal and tumor tissues were compared to elucidate their expression characteristics at the protein level.

### 2.7 Analysis of immune cell infiltration

The CIBERSORT algorithm was used to quantify the levels of immune cell infiltration between the mRNA NPC group and control groups on three mRNA training datasets: GSE12452, GSE13597, and GSE64634. A stacked bar chart was generated to visualize the relative abundance of each type of immune cell in each sample. Box plots were designed to compare the relative proportions of different immune cell types between the two groups. The “ggcorrplot” package was utilized to analyze the correlation between biomarkers and immune cell infiltration.

### 2.8 Prediction of potential targeted drugs

Using key target genes, potential therapeutic agents were screened using drug prediction tools (such as DGIdb) to design gene–drug interaction networks and identify candidate drugs with therapeutic potential for NPC.

### 2.9 Cell culture

We cultured the human NPC cell line C666-1 in complete medium containing 10% fetal bovine serum and 1% penicillin-streptomycin, and maintained the human nasopharyngeal epithelial cell line NP69 in an epithelial cell medium supplemented with specific growth factors. Both cell types were incubated at 37°C in an atmosphere of 5% CO_2_ and high humidity.

### 2.10 Quantitative reverse transcription polymerase chain reaction (RT-qPCR)

Human NPC cells and normal nasopharyngeal epithelial cells were harvested during the logarithmic growth phase. RNA was extracted using the TRIzol reagent and reverse-transcribed into cDNA. RT-qPCR experiments were performed using 2 × TB Green qPCR Master Mix. The thermal cycling protocol consisted of an initial denaturation step at 95°C for 30 seconds, followed by 40 cycles of denaturation at 95°C for 5 seconds and annealing/extension at 60°C for 34 seconds, and a melting curve analysis stage comprising 95°C for 15 seconds, 60°C for 1 min, and 95°C for 15 s. The relative expression levels of the target genes were calculated using the Livak method (2^^(-ΔΔCt)^), normalized to an internal control gene to account for inter-sample variation. All experiments were performed in triplicate. The primer sequences used are listed in Table 1.

**Table 1:** Primer design and sequence information.

Gene	Forward primer (5’ → 3’)	Reverse primer (5’ → 3’)
SOCS1	GCAGTCTCCACAGCAGCAGAGC	GGAATGTGCGGAAGTGCGTGT
β-actin	CGGGAAATCGTGCGTGAC	TGCCCAGGAAGGAAGGCT

SOCS1, suppressor of cytokine signaling 1

### 2.11 Ethics statement

All data in this study were obtained from publicly available databases (GEO and TCGA-HNSC). The original studies had received ethical approval from their respective institutional review boards; therefore, no additional ethical review or informed consent was required for this bioinformatic re-analysis.

## 3. Results

### 3.1 Data download results

The mRNA expression profile consists of three microarray datasets: GSE12452, GSE13597, and GSE64634. GSE12452 includes 10 non-nasopharyngeal carcinoma and 31 nasopharyngeal carcinoma tissue samples; GSE13597 includes 3 non-nasopharyngeal carcinoma and 25 nasopharyngeal carcinoma tissue samples; GSE64634 comprises 4 non-nasopharyngeal carcinoma and 12 nasopharyngeal carcinoma tissue samples; In contrast, the miRNA expression profile is based exclusively on the GSE32960 microarray dataset, which includes 16 non-nasopharyngeal carcinoma and 312 non-cancerous nasopharyngeal tissue samples (Table 2).

**Table 2:** Characteristics of mRNA and microRNA expression profiles in nasopharyngeal carcinoma.

GEO ID	Platform	Samples (N: T)	Expression profiling	Time
**GSE12452**	GPL570 [HG-U133_Plus_2] Affymetrix Human Genome U133 Plus 2.0 Array	10:31	mRNA	2008
**GSE13597**	GPL96 [HG-U133A] Affymetrix Human Genome U133A Array	3:25	mRNA	2009
**GSE64634**	GPL570 [HG-U133_Plus_2] Affymetrix Human Genome U133 Plus 2.0 Array	4:12	mRNA	2017
**GSE32960**	GPL14722 microRNA array	18:312	microRNA	2012

GEO, gene expression omnibus; N: normal; T: tumor.

### 3.2 Differential expression analysis of miRNA datasets

We identified 49 differentially expressed miRNAs in the GSE32960 dataset, as shown in a volcano plot and heatmap (Fig 1).

**Fig 1:**
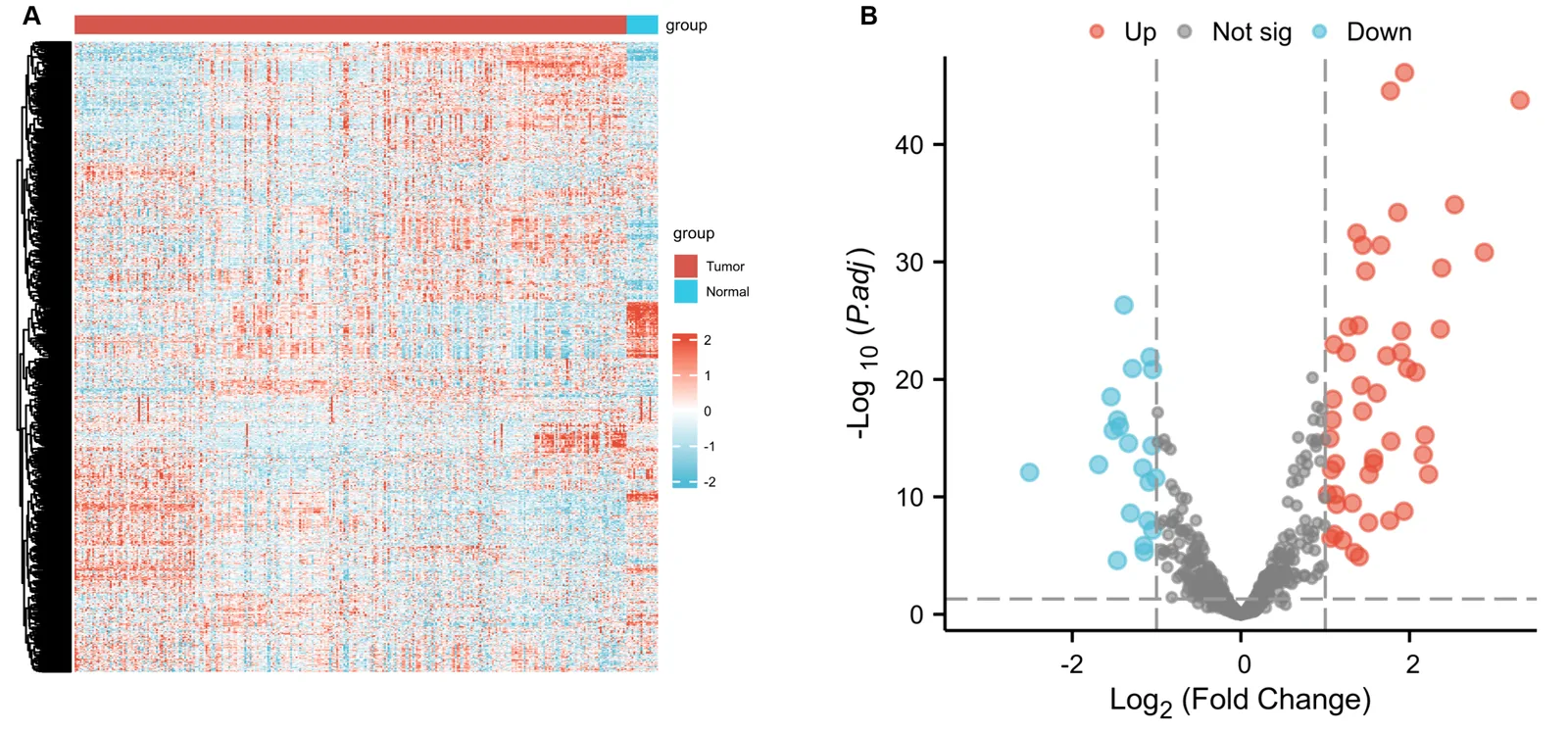
Differential expression analysis of miRNAs between nasopharyngeal carcinoma and normal tissues in the GSE32960 dataset. (A) miRNA differential expression heatmap. Red and blue denote high and low gene expression, respectively. In the top annotation bar, red represents the tumor group, and blue represents the normal group. (B) Volcano plot. Red dots indicate upregulated miRNAs, and blue dots indicate downregulated miRNAs; “Up”, “Down” and “Not sig” represent Up, upregulation; Down, downregulation; Not sig, no statistical significance.

### 3.3 Differential expression analysis of mRNA datasets

Integrating sequencing data from GSE12452, GSE13597, and GSE64634, we identified 1,050 differentially expressed mRNAs. Fig 2 shows the corresponding volcano plots and heat map.

**Fig 2:**
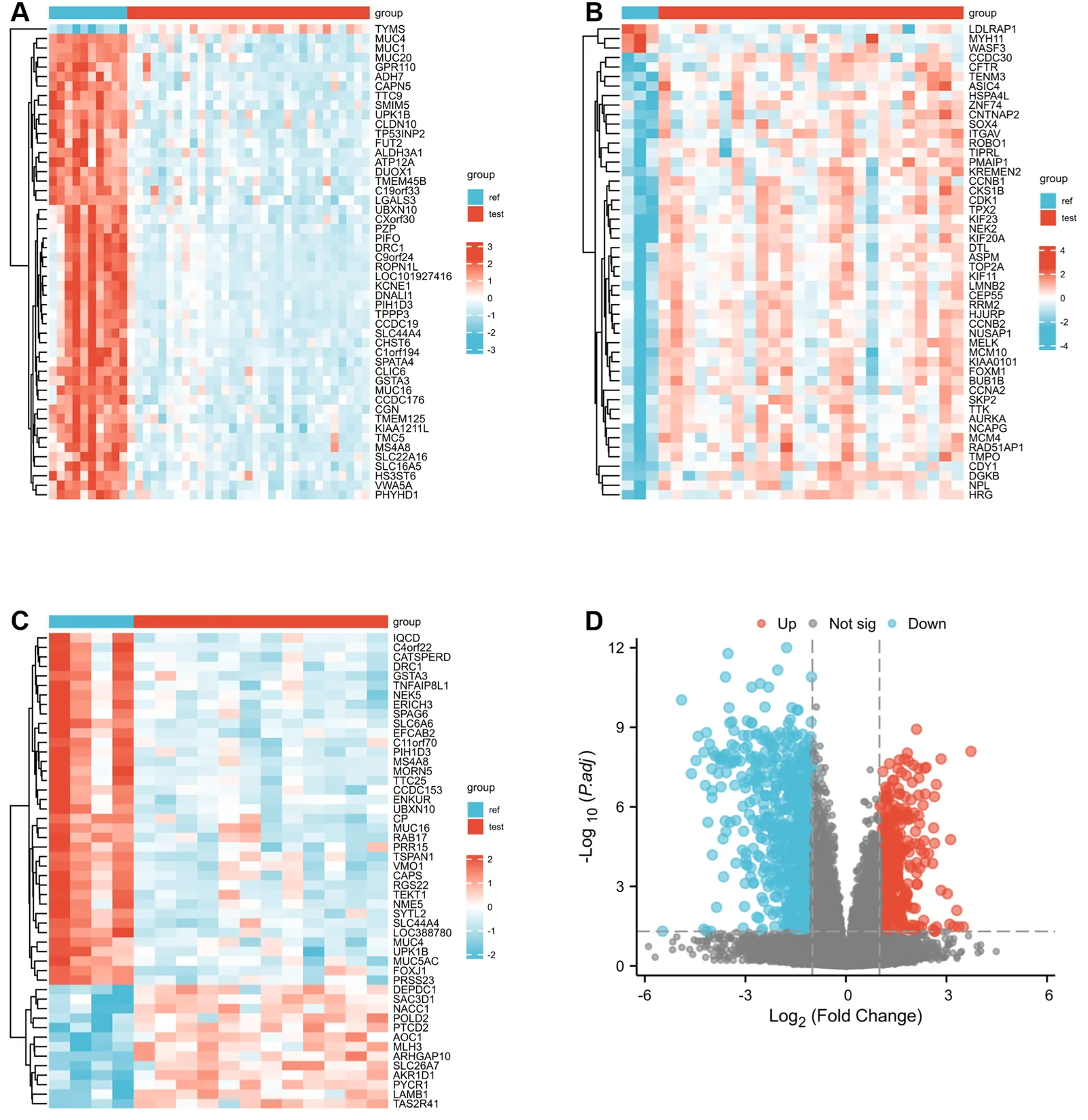
Analysis of differentially expressed genes in nasopharyngeal carcinoma and normal tissues from mRNA datasets. (A–C) Heat maps of differentially expressed genes in the GSE12452, GSE13597, and GSE64634 datasets, respectively. Red denotes high gene expression and blue denotes low gene expression. Ref, normal tissue group; Test, tumor tissue group. (D) Volcano plot of differentially expressed mRNAs after integrating the GSE12452, GSE13597, and GSE64634 datasets. Red dots indicate upregulated genes, and blue dots indicate downregulated genes; Up, upregulated; Down, downregulated; Not sig, not statistically significant.

### 3.4 Identification of miRNA target genes and screening of candidates

This study integrated three major databases—TargetScan, miRDB, and miRWalk—to conduct an in-depth analysis of potential downstream target genes of differentially expressed miRNAs, identifying 13,276, 5,399, and 4,682 candidate genes, respectively. Following the intersection of these datasets, 3,704 high-confidence target genes were identified. Subsequently, this gene set was cross-referenced with differentially expressed genes identified in the GSE12452, GSE13597, and GSE64634 datasets, yielding 213 shared genes. Building upon this result the gene set was subjected to a secondary screening against an immune-related gene dataset, ultimately identifying 17 core immune-related differentially expressed target genes (CXCL11, CCL8, SOCS1, ITGAV, PTGS2, RSAD2, TNFSF4, CCL2, AKT3, ROBO1, GHR, LGR5, NR4A2). The miRNA-target gene regulatory network constructed using Cytoscape software revealed that hsa-miR-30e-5p, hsa-miR-30a-5p, and hsa-miR-20a-5p exhibited the highest connectivity, regulat target in 625, 625, and 578 target genes, respectively. The results are presented in Fig 3.

**Fig 3:**
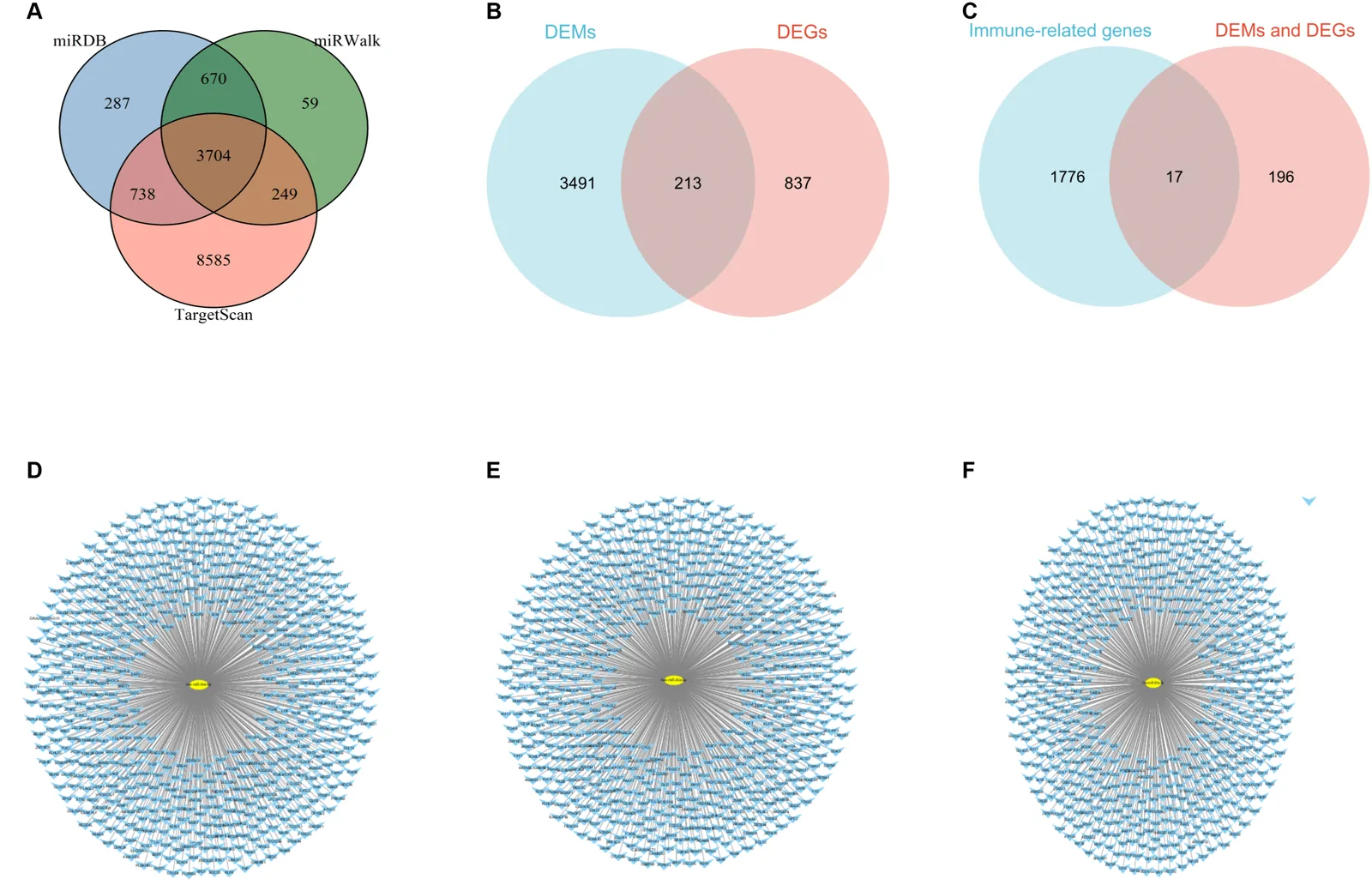
Screening of target genes and designing of the microRNA-mRNA regulatory network. (A) A Venn diagram showing the predictions of miRNA target genes from the three databases—miRDB, miRWalk, and TargetScan—with a total of 3,704 target genes in their intersection;(B) Venn diagram depicting the intersection of between differentially expressed microRNAs (DEMs) and differentially expressed genes (DEGs); (C) Venn diagram illustrating the common genes shared among immune-related genes, DEMs and DEGs; (D–F) Regulatory networks illustrating miRNA–target gene interactions for hsa-miR-30e-5p, hsa-miR-30a-5p, and hsa-miR-20a-5p.

### 3.5 GO and KEGG enrichment analysis for candidate genes

In this study, GO functional annotation and KEGG pathway enrichment analyses were performed on 17 core immune-related differentially expressed target genes (as shown in Fig 4A and 4B). The GO enrichment results indicated that the enriched biological processes primarily included cytokine-regulated signaling pathways, chemokine response, and janus kinase (JAK) activation. Molecular functions were predominantly characterized by the binding activity of cytokines, chemokines, and their receptors. KEGG pathway analysis revealed that these genes were significantly enriched in immune-related pathways, including cytokine-receptor interactions and the JAK-STAT signaling pathway, and were also involved in fluid shear stress and the prolactin signaling pathway. These findings indicate that the core target genes may contribute to the development and malignant progression of NPC by modulating multiple immune-related pathways.

**Fig 4:**
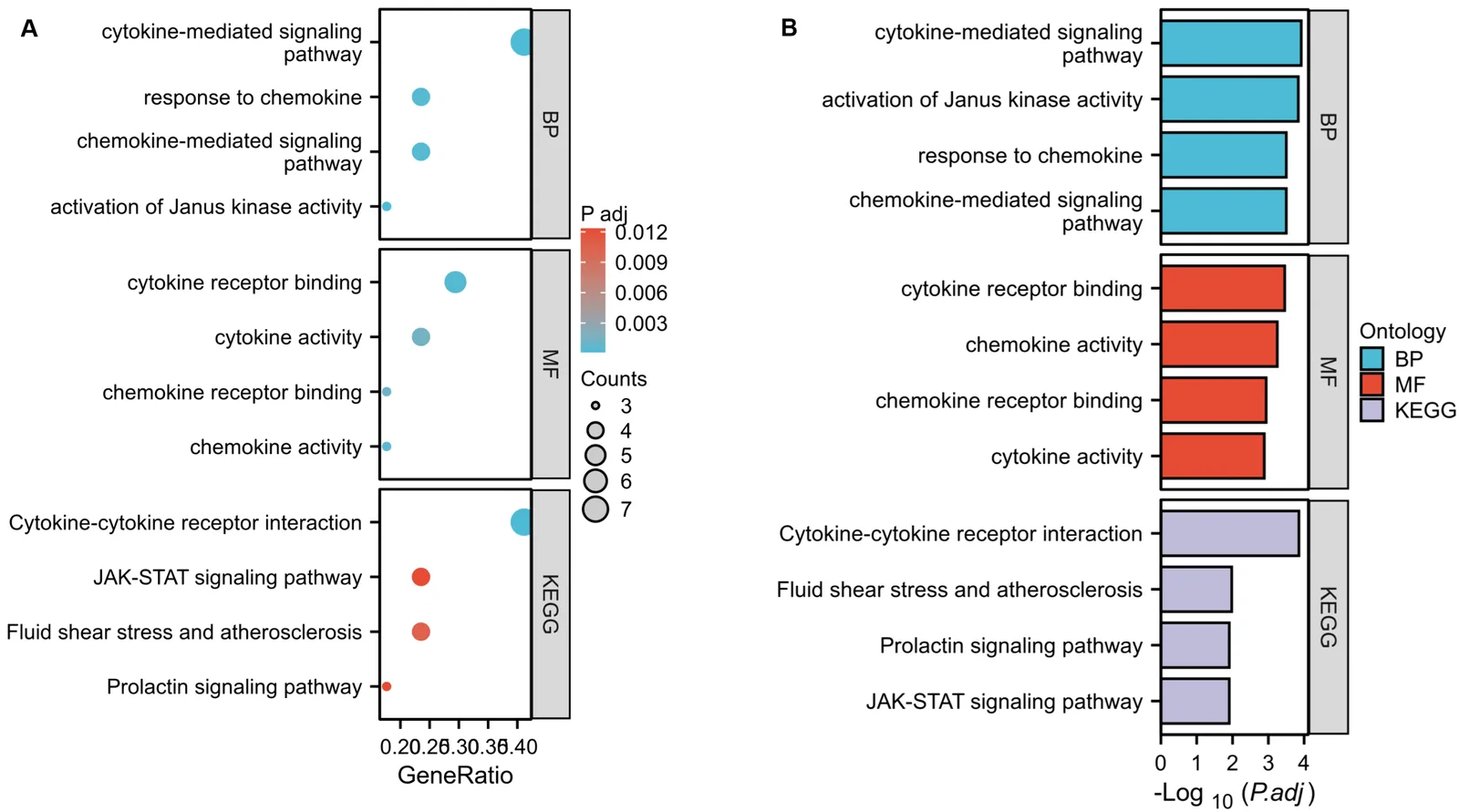
GO and KEGG enrichment analysis of candidate genes. (A) Bubble plot of the GO and KEGG enrichment results; bubble size represents the number of enriched genes, and color intensity indicates the adjusted P-value. (B) Bar charts of the GO and KEGG enrichment results. GO, Gene ontology; BP, Biological Process; MF, Molecular Function; KEGG, Kyoto Encyclopedia of Genes and Genomes pathways. JAK-STAT, janus kinase signal transducer and activator of transcription.

### 3.6 Analysis of PPI networks and co-expression correlations for candidate key genes

To investigate the functional relationships and co‑expression patterns among the 17 candidate key genes, we constructed a protein‑protein interaction (PPI) network and an expression correlation heatmap. The PPI network derived from the STRING database (Fig 5A) revealed that the 17 key genes did not function independently but formed a well‑structured, tightly connected interaction network. Genes such as SOCS1, CCL2, PTGS2, CXCL11, and RSAD2 served as hub nodes in the network and interacted directly with many other genes, suggesting that these genes may play key regulatory roles in common biological pathways. To further validate the co‑regulatory relationships among these key genes at the expression level, we generated an expression correlation heatmap of the 17 genes using samples from the training set (Fig 5B). Most key genes exhibited significant positive correlations (P < 0.05), corresponding to the large red areas in the heatmap. No significant strong negative correlations were observed, with only a few gene pairs showing weak correlations or no statistical significance.

**Fig 5:**
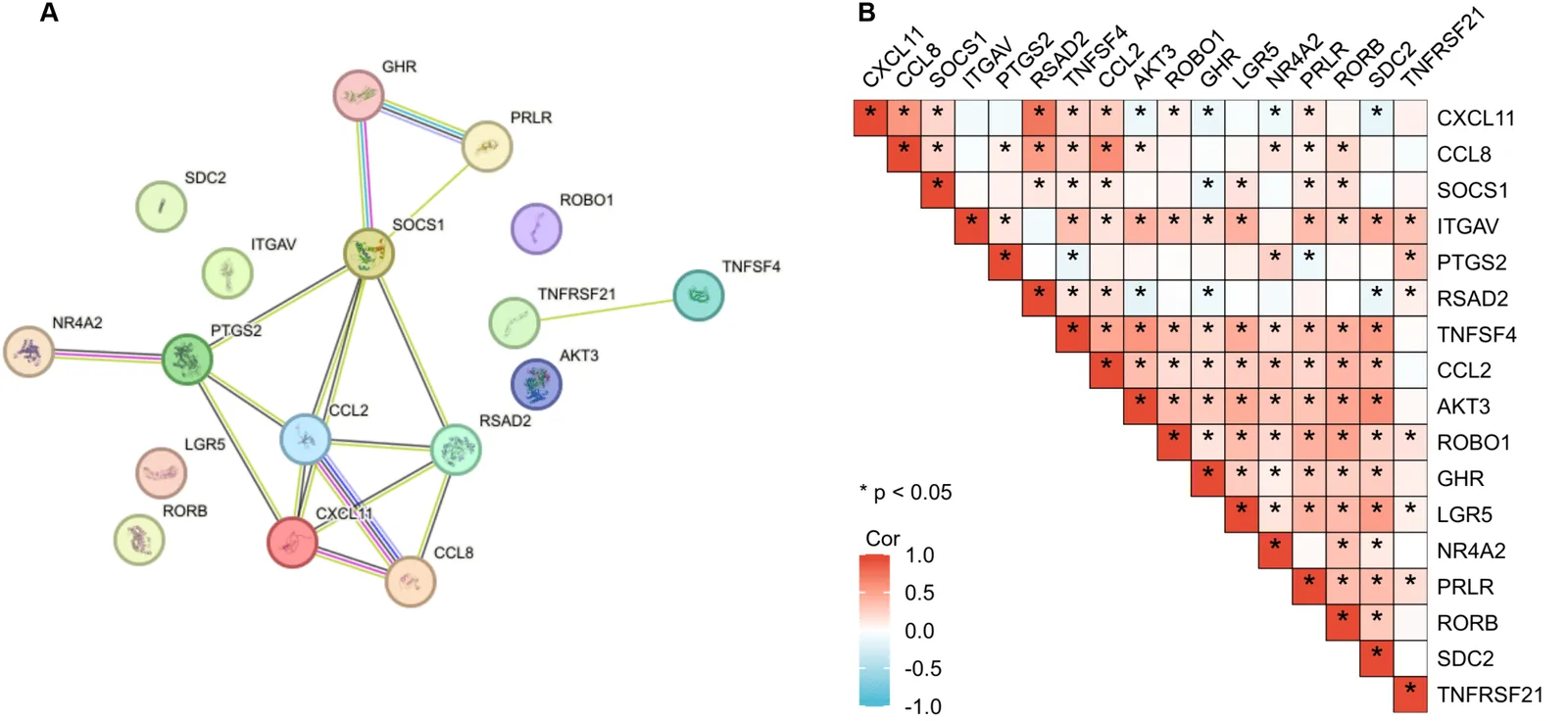
Analysis of protein-protein interaction (PPI) networks and gene-gene correlations of candidate genes. (A) Protein-protein interaction (PPI) network diagram for candidate genes; the lines represent interactions between genes. (B) Heat map showing correlations among candidate genes; red indicates a positive correlation, blue indicates a negative correlation, and P < 0.05 is marked with an asterisk (*).

### 3.7 Construction and validation of a prognostic risk model for NPC

To further identify key regulatory genes driving the onset and progression of nasopharyngeal carcinoma, this study utilized the TCGA Head and Neck Squamous Cell Carcinoma (HNSC) dataset, focusing on 17 differentially expressed immune-related genes, and employed the LASSO Cox regression algorithm to construct a prognostic risk model. Through cross-validation, the optimal lambda value (lambda.min = 0.029597) was determined and variables were screened, ultimately identifying five prognosis-related genes: CXCL11, SOCS1, ROBO1, LGR5, and RORB. Based on the regression coefficients of each gene, a prognostic risk score formula for NPC was developed as follows: risk score = (−0.18157) × CXCL11 + (−0.29542) × SOCS1 + (−0.154815) × ROBO1 + (−0.18165) × LGR5 + (−0.14569) × RORB. To evaluate the model’s prognostic predictive performance, this study plotted time-dependent ROC curves for predicting 1-year, 3-year, and 5-year overall survival (OS), with corresponding areas under the curve (AUC) of 0.574, 0.622, and 0.636, respectively. Subsequently, patients were divided into high-risk and low-risk groups based on the optimal risk threshold. Kaplan-Meier survival analysis showed that the overall survival time of patients in the high-risk group was significantly shorter than that of the low-risk group (P < 0.001, HR = 1.65). as shown in Fig 6.

**Fig 6:**
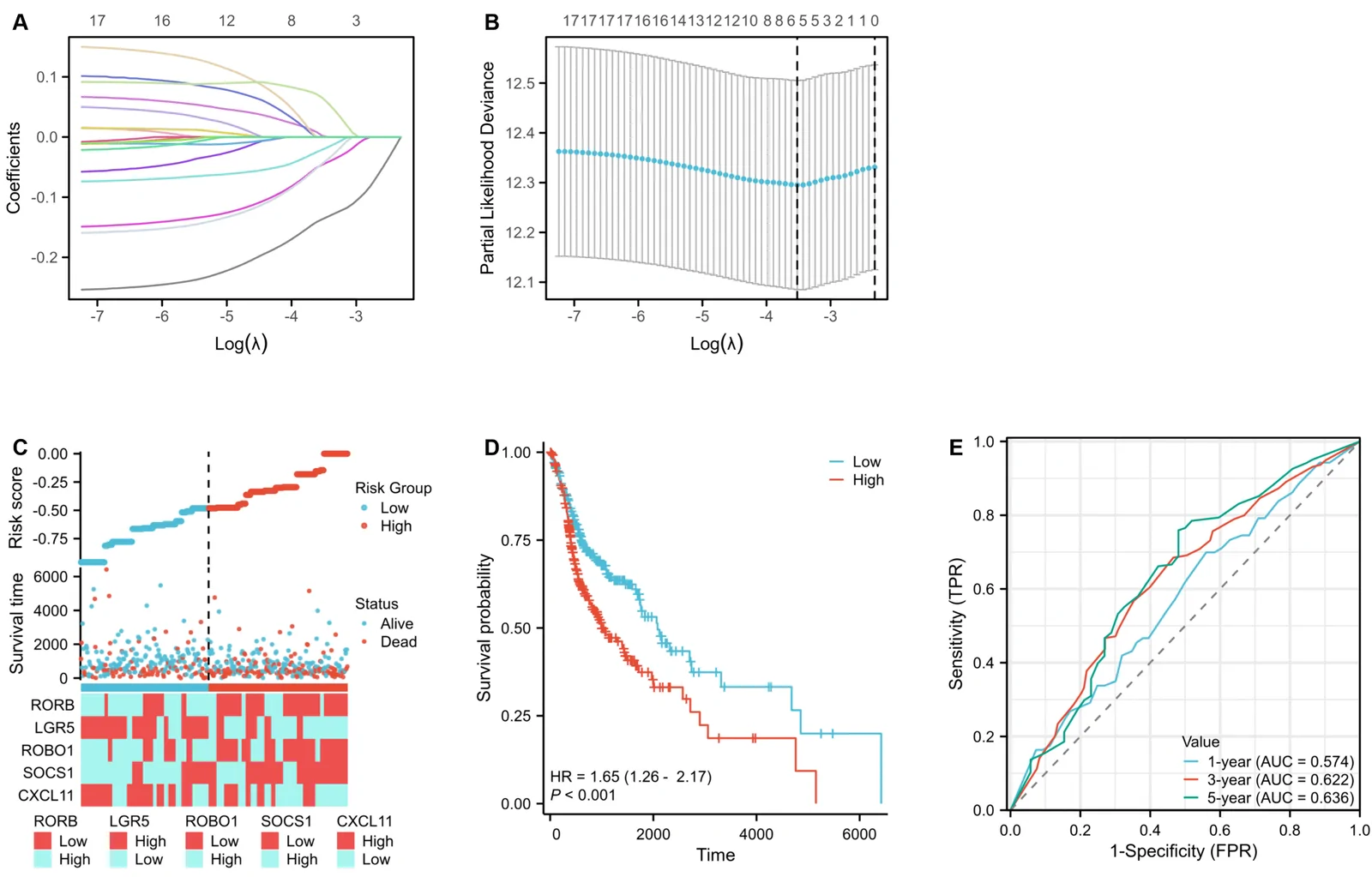
Building a prognostic risk model based on LASSO-Cox regression. (A) LASSO regression coefficient change curve; the numbers above represent the number of genes included in the model; (B) Determine the optimal value of λ based on the partial likelihood bias; the dashed line marks the position corresponding to the optimal λ; (C) Risk score distribution, patient survival status, and gene expression heatmap for the model; (D) Kaplan-Meier survival curves for the high- and low-risk groups; HR stands for hazard ratio; (E) Time-dependent ROC curves for the model’s 1-year, 3-year, and 5-year survival predictions.

### 3.8 Analysis of immune cell infiltration

To investigate the characteristics of immune cell infiltration in the nasopharyngeal carcinoma microenvironment and the associations between key genes and immune cells, this study systematically analyzed the immune cell infiltration profile and the correlations between key genes and immune cells based on three mRNA training datasets: GSE12452, GSE13597, and GSE64634. Analysis of immune cell infiltration revealed extensive and heterogeneous infiltration of immune cells in the nasopharyngeal carcinoma tumor microenvironment, primarily consisting of macrophages, T cell subsets, and neutrophils (Fig 7A). Compared with normal tissues, there were significant differences in the infiltration levels of various immune cells in tumor tissues, suggesting an imbalance in the immune microenvironment (Fig 7B). Correlation analysis between key genes and immune cell infiltration indicated that the expression levels of CXCL11, SOCS1, ROBO1, LGR5, and RORB were significantly positively correlated with the infiltration levels of various immune cells, including CD8 + T cells, macrophages, and neutrophils (P < 0.05, Fig 7C).

**Fig 7:**
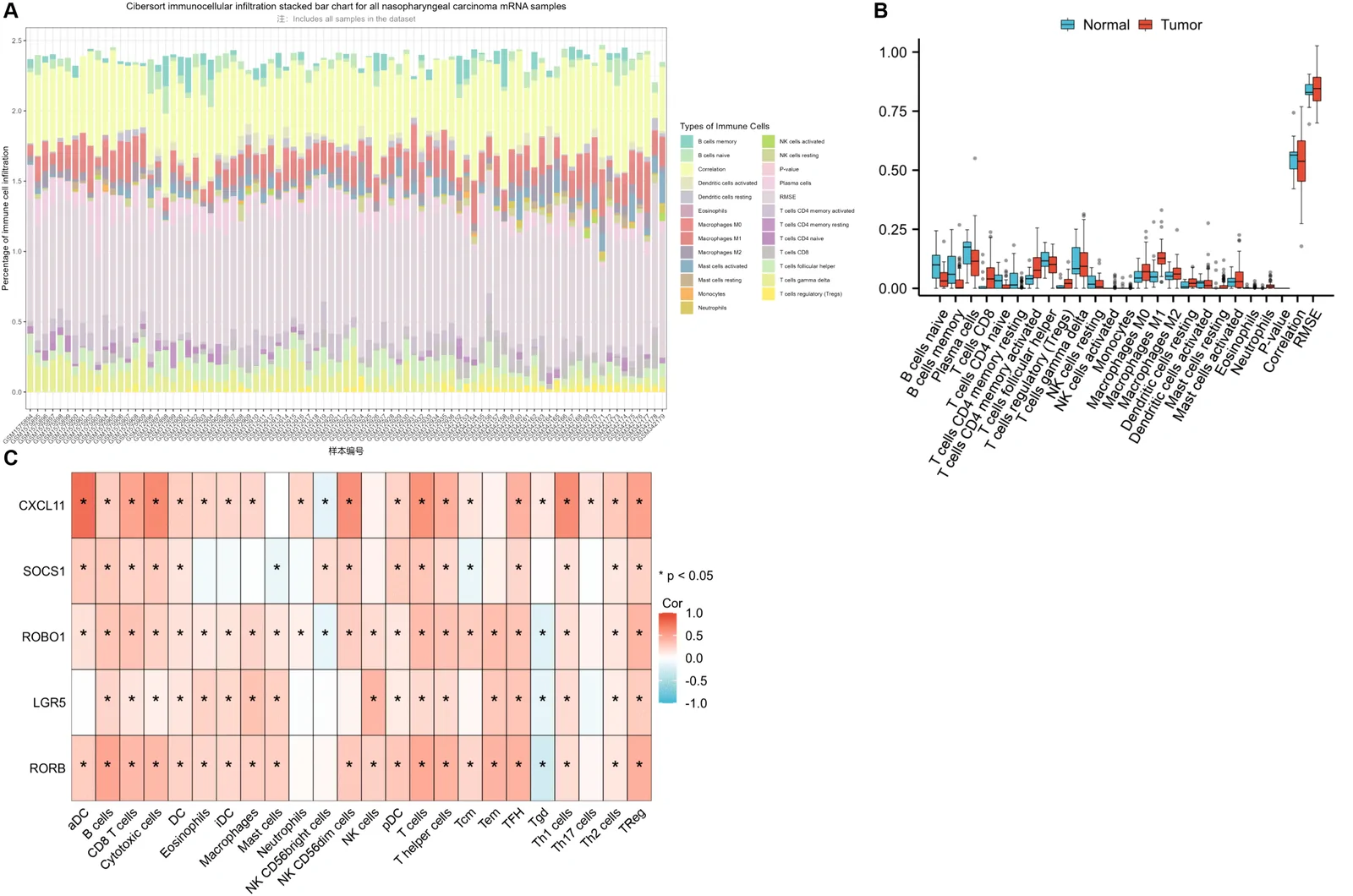
Analysis of immune cell infiltration in nasopharyngeal carcinoma and association analysis between prognostic risk genes and immune cells. (A) A stacked bar chart showing the estimated proportions of various immune cell infiltrates in all nasopharyngeal carcinoma samples, as calculated by the CIBERSORT algorithm; (B) Box-and-whisker plots showing the levels of infiltration of 22 types of immune cells in normal tissue and nasopharyngeal carcinoma tissue; blue represents the normal group, and red represents the tumor group; (C) Heatmap showing the correlation between five prognostic risk genes and the level of immune cell infiltration; red indicates a positive correlation, blue indicates a negative correlation, and P* < 0.05.

### 3.9 Evaluation of the diagnostic performance of key genes in the NPC training set

To further evaluate the potential value of these five key genes as diagnostic biomarkers for nasopharyngeal carcinoma, this study conducted receiver operating characteristic (ROC) curve analyses using the TCGA head and neck squamous cell carcinoma (HNSC) training set (Fig 8A–8E). The results showed that SOCS1 demonstrated extremely high diagnostic accuracy (AUC = 0.935, 95% CI: 0.902–0.969) and CXCL11 exhibited high diagnostic performance (AUC = 0.809, 95% CI: 0.746–0.873), with both indicating significant diagnostic potential, whereas RORB (AUC = 0.673) and LGR5 (AUC = 0.654) demonstrated moderate diagnostic performance; in contrast, ROBO1 exhibited low diagnostic accuracy (AUC = 0.473), with no statistically significant difference compared with random chance (AUC = 0.5).

**Fig 8:**
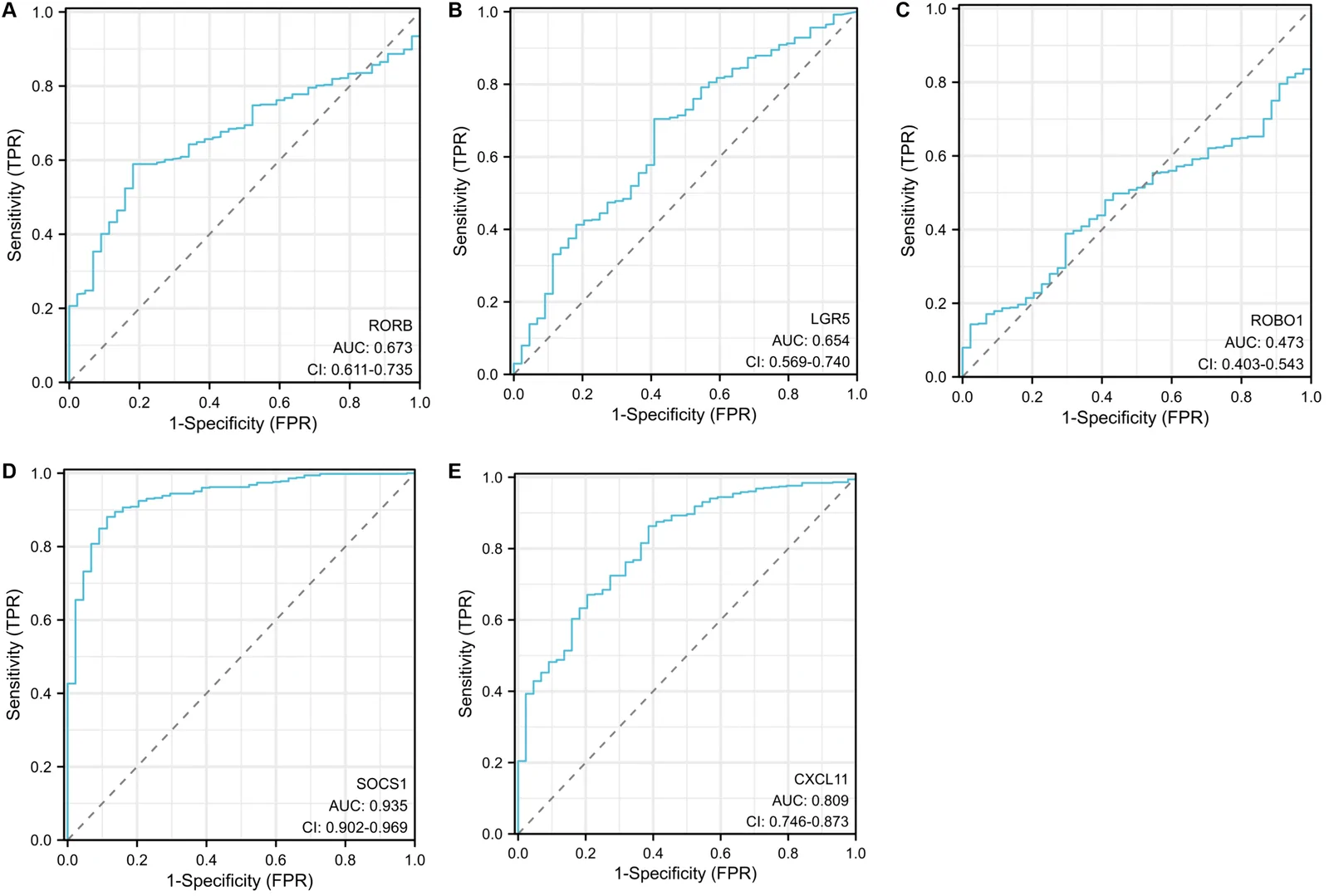
Receiver operating characteristic (ROC) curves illustrating the diagnostic value of five biomarkers in the nasopharyngeal carcinoma training dataset. (A–E) Receiver operating characteristic (ROC) curves for the candidate diagnostic biomarkers RORB, LGR5, RORC, SOCS1, and CXCL1 in training cohort. AUC, area under the curve; CI, confidence interval; TPR, true predictive rate; FPR, false predictive rate.

### 3.10 Differential expression of key genes in HNSC tumor versus normal tissues

Based on the TCGA-HNSC cohort, this study compared the expression levels of RORB, LGR5, ROBO1, SOCS1, and CXCL11 in 44 normal tissue samples and 520 primary tumor tissue samples. The results (Fig 9A-9E) showed that, Compared with normal tissues, RORB was significantly downregulated in tumor tissues (P < 0.001), while LGR5 was significantly upregulated (P < 0.01); ROBO1, SOCS1, and CXCL11 were also significantly upregulated in tumor tissues, with P-values of 0.001, 0.0001, and 0.001, respectively.

**Fig 9:**
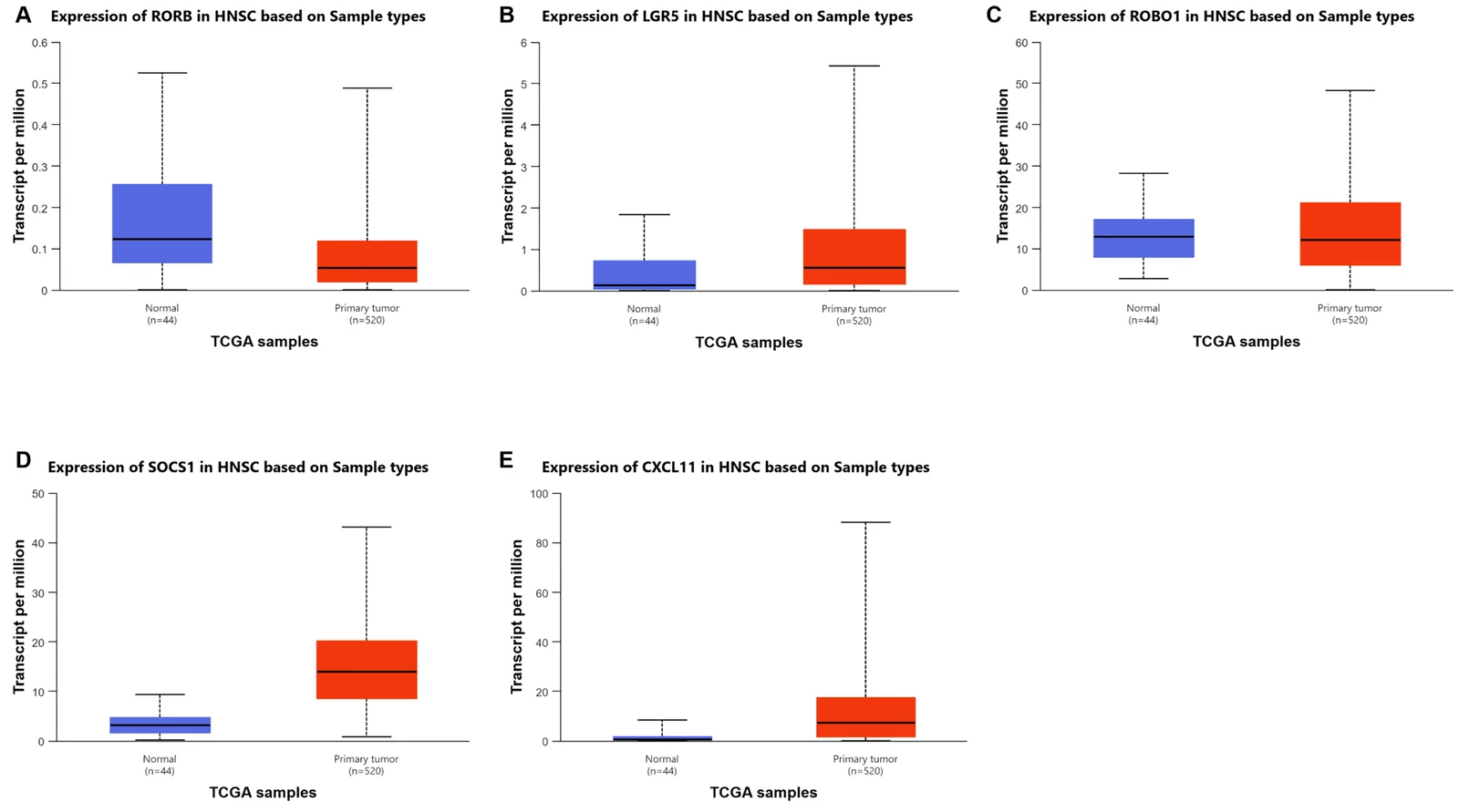
Box plots showing the expression levels of five key genes in normal and tumor tissues from the TCGA dataset. (A–E) show in order, the expression levels of RORB, LGR5, ROBO1, SOCS1, and CXCL11 respectively, in the normal group (n = 44) and the primary tumor group (n = 520).

### 3.11 Predictive value of key gene expression for 1-year survival in NPC

To evaluate the predictive performance of five key genes—RORB, LGR5, ROBO1, SOCS1, and CXCL11—on 1-year overall survival in nasopharyngeal carcinoma patients, this study plotted receiver operating characteristic (ROC) curves for 1-year overall survival (OS) based on the HNSC training set. The results (Fig 10A-10E) showed that the predictive performance of all five genes was unsatisfactory (AUCs all < 0.6): the AUCs for RORB, LGR5, ROBO1, and SOCS1 were 0.406, 0.382, 0.466, and 0.422, respectively, with values generally close to or below the random level (AUC = 0.5), indicating low predictive accuracy; The AUC for CXCL11 was 0.524; although slightly higher than the random level, its overall predictive value remained limited. These results indicate that individual genes are unlikely to serve as independent predictors of 1-year survival in patients with NPC, further supporting the superior predictive value of multigene risk scoring models in prognostic assessment.

**Fig 10:**
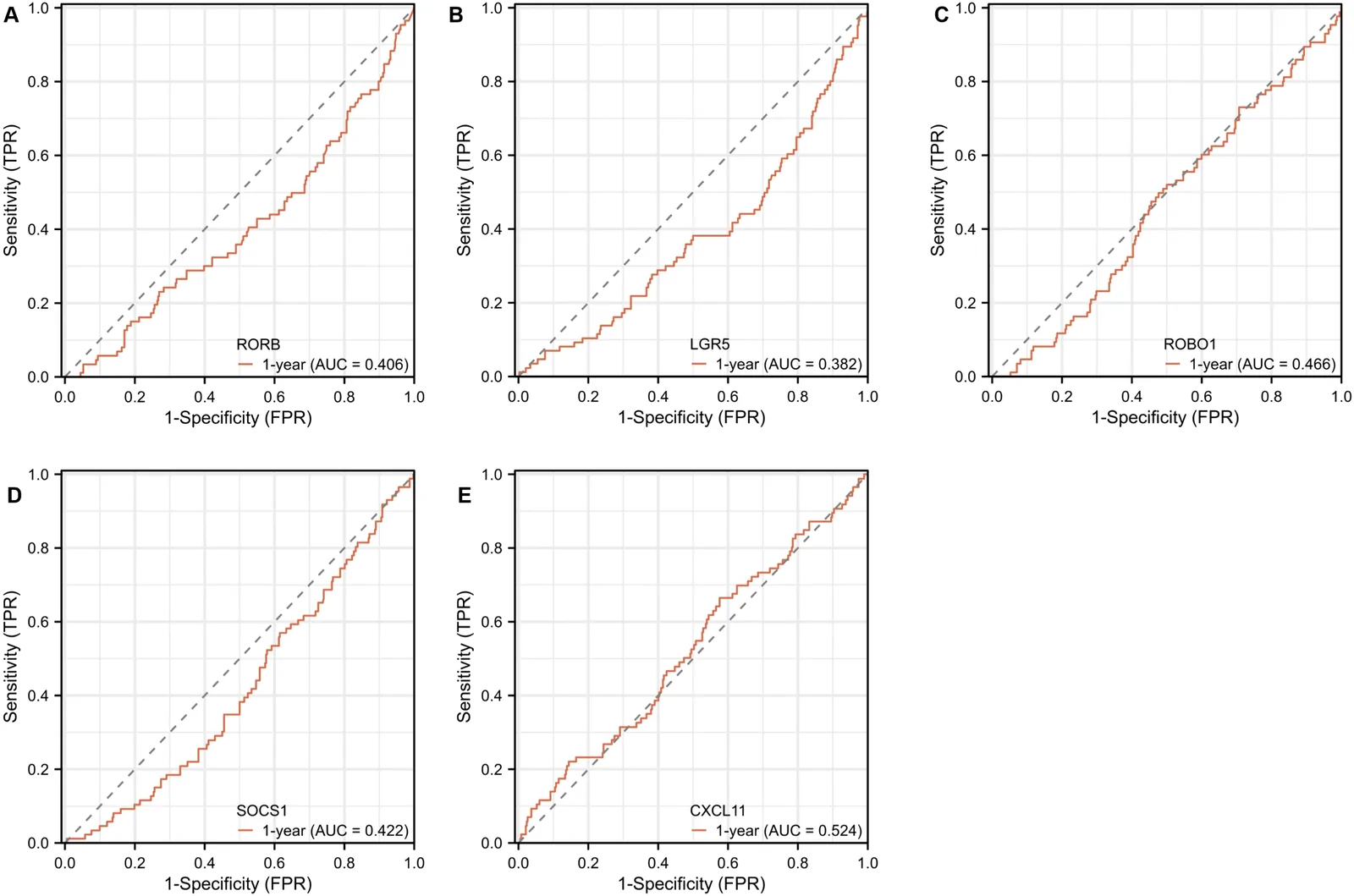
ROC curves showing the predictive performance of five key genes for 1-year prognosis. (A–E) Receiver operating characteristic (ROC) curves for RORB, LGR5, ROBO1, SOCS1, and CXCL11 in predicting 1-year survival status.

### 3.12 Validation of protein expression of key genes in NPC and normal tissues

To investigate differences in protein expression levels of the five key genes—RORB, LGR5, ROBO1, SOCS1, and CXCL11—this study utilized the HPA database to analyze immunohistochemical results from head and neck cancer tissues (using nasopharyngeal carcinoma as an example) and normal nasopharyngeal tissues. The results showed (Fig 11A-11E) that RORB and SOCS1 were downregulated in tumor tissue, LGR5 was upregulated, ROBO1 exhibited a specific alteration in its expression pattern, and no significant differences in expression levels were observed for CXCL11. These protein expression findings further support the reliability of these genes as potential biomarkers for NPC.

**Fig 11:**
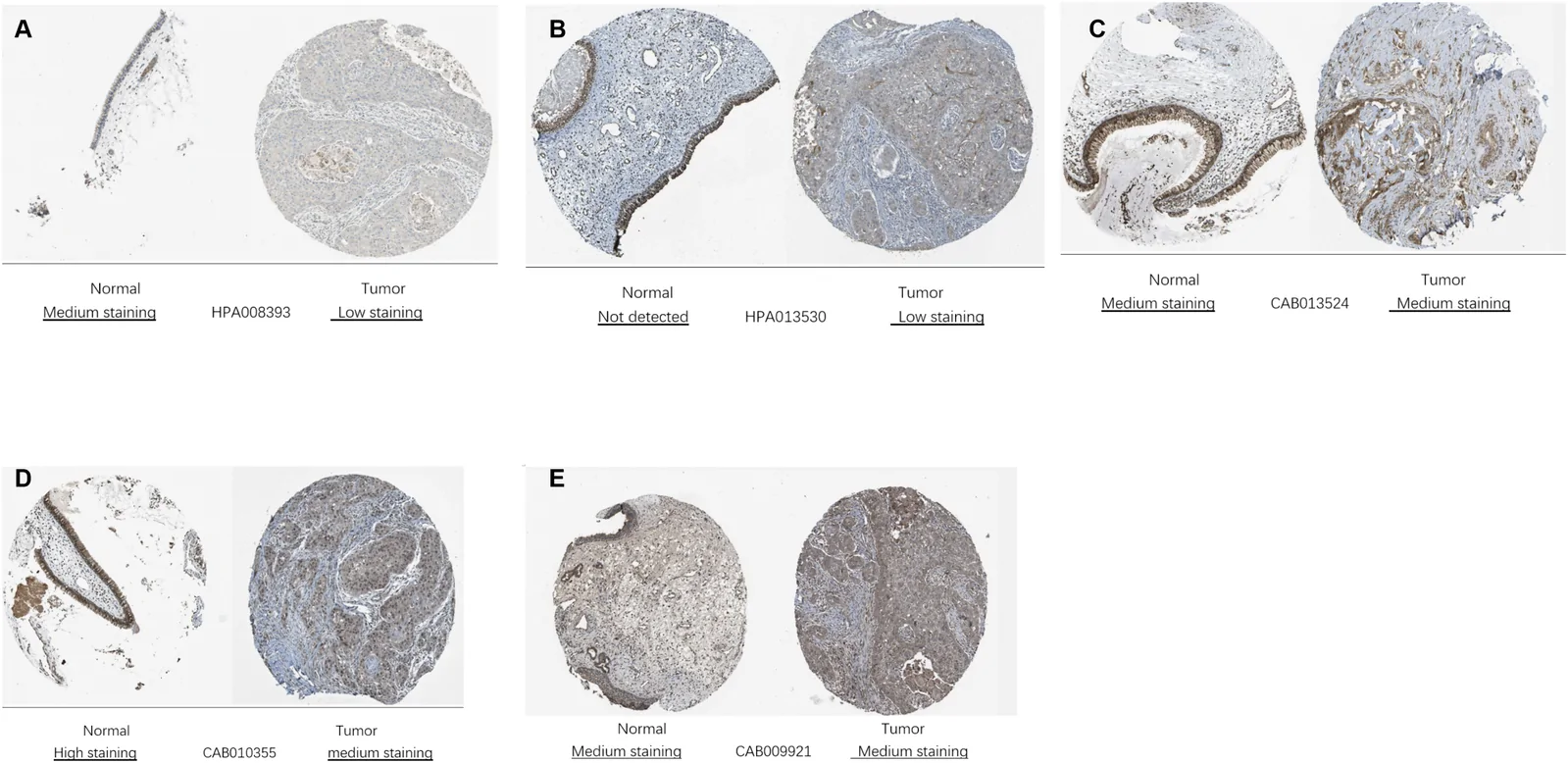
Immunohistochemical staining images of 5 candidate genes from the Human Protein Atlas database. (A–E) Representative immunohistochemical sections for RORB, LGR5, ROBO1, SOCS1, and CXCL11, respectively; each group shows staining results for paired normal and tumor tissues. The corresponding antibody codes and staining grades (including no expression, low expression, moderate expression, and high expression) are labeled below each section.

### 3.13 Prediction of drug-gene interactions

This study identified potential targeted drugs based on the key targets SOCS1 and LGR5. Among these, fluorouracil targets LGR5, while aldesleukin targets the SOCS1 gene; these findings provide a preliminary basis for drug screening in the development of targeted therapies for nasopharyngeal carcinoma. The results are visualized in Fig 12.

**Fig 12:**
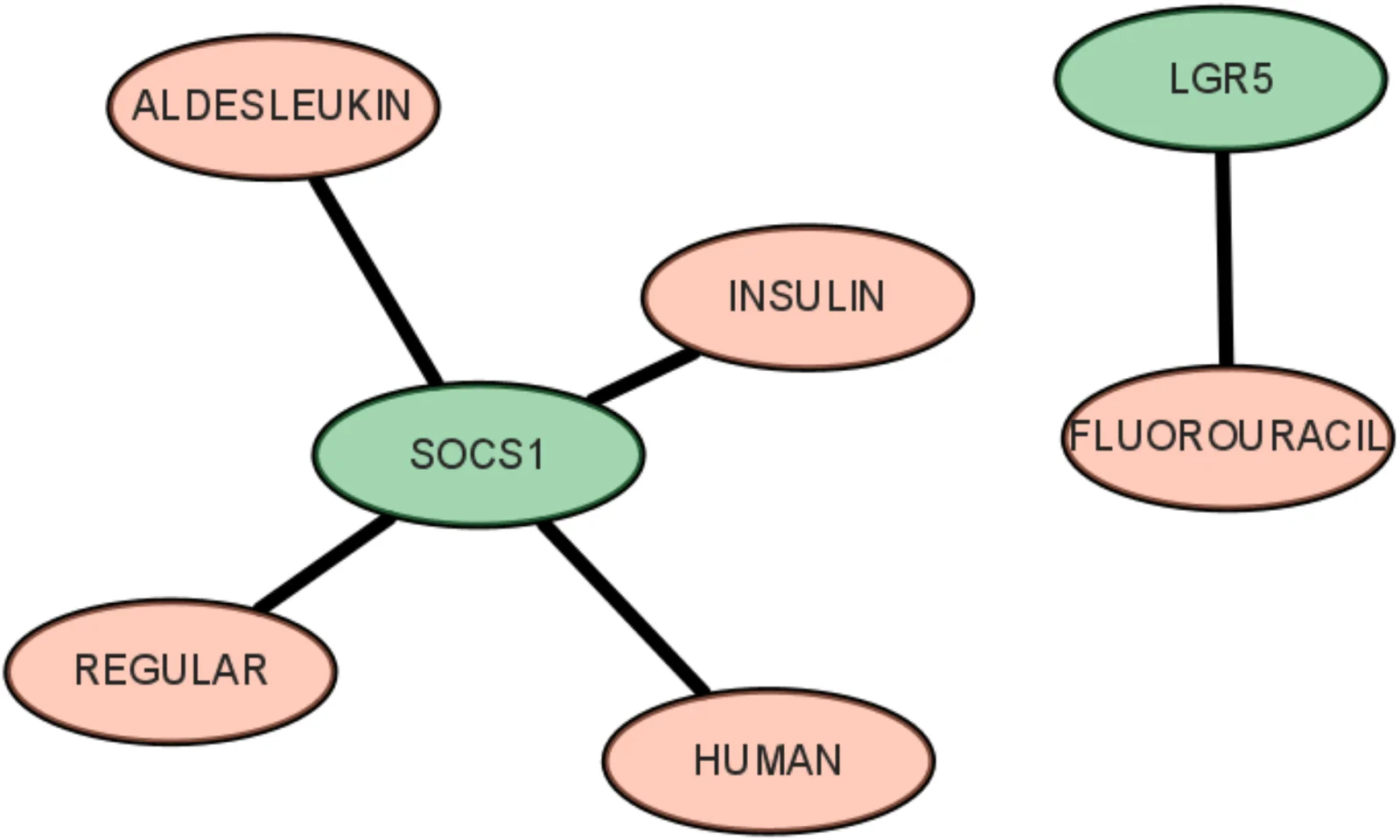
Predicted network of potential therapeutic drugs for the core genes SOCS1 and LGR5. (Green ovals represent core target genes, while pink ovals represent candidate small-molecule drugs and biologics that have a targeted regulatory relationship with the core target genes.).

### 3.14 RT-qPCR results

RT-qPCR results (Fig 13) showed significantly elevated SOCS1 mRNA expression levels in NPC cells compared with those in normal nasopharyngeal epithelial tissue (P < 0.01). The relative expression level was approximately 3.7 times that in the normal control group.

**Fig 13:**
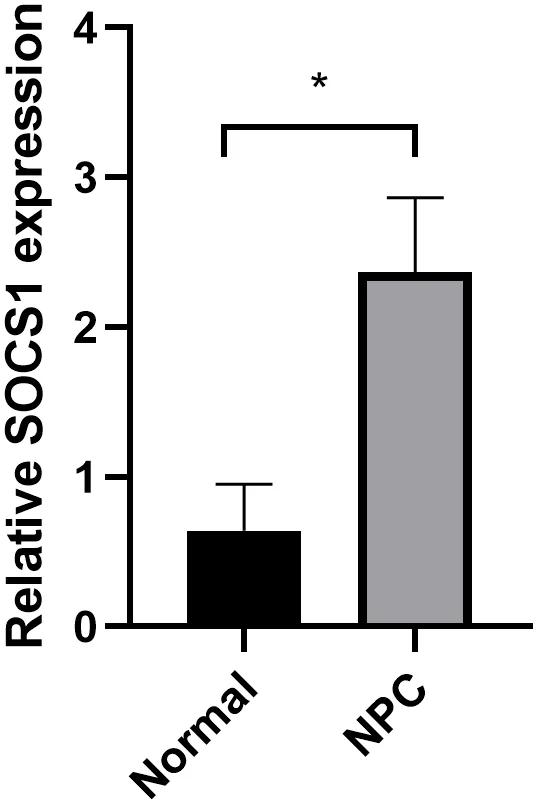
Relative mRNA expression levels of the SOCS1 gene in nasopharyngeal carcinoma (NPC) and normal tissues. Note, *P < 0.05. Normal, Human nasopharyngeal epithelial tissue, NPC, Nasopharyngeal carcinoma tissue.

## 4. Discussion

Nasopharyngeal carcinoma has become one of the most common malignant tumors of the head and neck, with an incidence rate in East and Southeast Asia that is significantly higher than in other regions of the world [21]. In recent years, the overall incidence of nasopharyngeal carcinoma has shown a continuous upward trend. Patients with advanced nasopharyngeal carcinoma are prone to local recurrence and distant metastasis [22], and their treatment options are very limited. With the rapid advancement of multi-omics research, our understanding of the pathogenesis of different molecular subtypes of nasopharyngeal carcinoma has greatly improved, and the discovery of new biomarkers for nasopharyngeal carcinoma has brought hope for precision therapy in advanced cases [23]. In this study, by integrating miRNA and mRNA multi-omics expression data, we constructed a miRNA-mRNA regulatory network and focused on core immune-related genes to investigate their roles and potential molecular mechanisms in nasopharyngeal carcinoma progression, with the aim of identifying new therapeutic targets for immunotherapy against nasopharyngeal carcinoma.

By integrating miRNA-mRNA regulatory networks with analyses of immune-related genes, our study revealed the potential roles of three key miRNAs (hsa-miR-30e-5p, hsa-miR-30a-5p, and hsa-miR-20a-5p) in the prognosis of nasopharyngeal carcinoma and further identified 17 core immune-related differentially expressed target genes. These genes are significantly enriched in cytokine-mediated signal transduction, chemokine response, and the JAK-STAT signaling pathway, indicating that immune regulatory mechanisms play a critical role in the progression of nasopharyngeal carcinoma. A prognostic model constructed based on the five key genes—CXCL11, SOCS1, ROBO1, LGR5, and RORB—demonstrated good diagnostic performance in nasopharyngeal carcinoma, providing a new direction for precise prognostic assessment of the disease. The five identified key genes (CXCL11, SOCS1, ROBO1, LGR5, and RORB) were all significantly positively correlated with the infiltration levels of various immune cells, including CD8 ⁺ T cells, macrophages, and neutrophils, suggesting that these genes may influence the progression of nasopharyngeal carcinoma by regulating the recruitment and activation of immune cells in the tumor microenvironment. Based on drug predictions from DGIdb, we found that 5-fluorouracil targets LGR5 and aldesleukin targets SOCS1, providing preliminary drug candidates for targeted therapy in nasopharyngeal carcinoma. As a classic chemotherapeutic agent, 5-fluorouracil may inhibit tumor stem cell activity by targeting LGR5 through the Wnt/β-catenin signaling pathway; aldesleukin (an IL-2 analog) may enhance T-cell-mediated antitumor immune responses by regulating SOCS1. These findings provide a direction for subsequent functional experiments and clinical translation. RT-qPCR analysis in this study revealed that SOCS1 mRNA expression levels in nasopharyngeal carcinoma tissues were significantly upregulated compared to normal nasopharyngeal epithelial tissues, approximately 3.7-fold higher than the normal control group (P < 0.01), suggesting that SOCS1 may play a significant role in the development and progression of nasopharyngeal carcinoma. SOCS1 is a key member of the cytokine signal-suppressing kinase family and primarily regulates cell proliferation, differentiation, and immune responses by negatively modulating the JAK/STAT pathway [24]. Previous studies have generally regarded SOCS1 as a potential tumor suppressor gene; in many tumors, its expression is often silenced due to promoter methylation, leading to sustained activation of the JAK/STAT pathway and thereby promoting tumor initiation and progression [25]. However, the results of this study show that SOCS1 is significantly upregulated in nasopharyngeal carcinoma cells, differing from the traditional role of tumor suppressor genes, suggesting that its function in nasopharyngeal carcinoma may exhibit tissue specificity and functional heterogeneity. This finding may be related to the unique etiological background of nasopharyngeal carcinoma. EBV infection is one of the key factors in the development of nasopharyngeal carcinoma; proteins encoded by EBV, such as LMP1, can induce the upregulation of SOCS1 through feedback mechanisms by activating signaling pathways including NF-κB and STAT [26–28]. In this context, highly expressed SOCS1 may not exert its traditional tumor-suppressor function but rather participate in regulating inflammatory responses and immunosuppressive states within the tumor microenvironment, thereby aiding tumor cells in evading immune surveillance [29,30]. Furthermore, some studies suggest that in certain tumors, SOCS1 may exhibit oncogenic properties by inhibiting the interferon signaling pathway, thereby reducing the sensitivity of tumor cells to immune attack [31,32].

In summary, through comprehensive bioinformatics analysis, we identified five key genes—CXCL11, SOCS1, ROBO1, LGR5, and RORB—closely associated with the prognosis of NPC. Among them, SOCS1 plays a critical role in immune-related biological processes in NPC, suggesting its potential as a biomarker for prognostic assessment and prediction of immunotherapy response in NPC patients. Nevertheless, several limitations should be acknowledged. First, the prognostic risk model was constructed and validated based on retrospective publicly available database cohorts, and prospective clinical cohorts are needed to further validate its clinical predictive performance. Second, although our in vitro experiments included both NPC cell lines and normal nasopharyngeal epithelial cells, in vivo animal studies are still lacking to verify the biological functions of these key genes. Third, the miRNA–mRNA regulatory network constructed in this study was based solely on bioinformatics predictions; further mechanistic investigations, including additional in vitro functional experiments, are required to elucidate the precise molecular interactions. Overall, we have interpreted the observational data with caution and refrained from overinterpretation.

## 5. Conclusion

By analyzing multiple gene-expression profiles as well as the TCGA-HNSC dataset, we screened five genes potentially linked to NPC development, which exert important immunological functions during tumor progression and metastasis. Our results also indicated that SOCS1 represents a promising biomarker for NPC patients and may serve as a predictor for prognosis and immunotherapy response.

## Supporting information

S1 File R script for bioinformatics analysis of nasopharyngeal carcinoma prognosis. (R)
